# Levels of age‐related blood CD8 T cells binding a non‐self peptide/HLA epitope track with Alzheimer's disease status in HLA‐A2^+^ and HLA‐A2^‐^ cohorts: a T cell biomarker assay applicable to all patients

**DOI:** 10.1002/alz70856_107315

**Published:** 2026-01-09

**Authors:** Christopher J. Wheeler, Debby Van Dam, Yannick Vermeiren, Hans De Reu, Peter Paul De Deyn

**Affiliations:** ^1^ Brain Mapping Foundation, Soc. for Brain Mapping & Therapeutics, Pacific Palisades, CA, USA; ^2^ T‐Neuro Pharma, Inc., Albuquerque, NM, USA; ^3^ University of California, Santa Cruz, Santa Cruz, CA, USA; ^4^ StemVax Therapeutics, Los Angeles, CA, USA; ^5^ Reference Center for Biological Markers of Dementia (BIODEM), Laboratory of Neurochemistry and Behavior, Institute Born‐Bunge, University of Antwerp, Antwerp, Belgium; ^6^ University of GrÖningen, University Medical Center Groningen (UMCG), Department of Neurology and Alzheimer Research Center, GrÖningen, Netherlands; ^7^ Division of Human Nutrition and Health, Wageningen University & Research, Wageningen, Netherlands; ^8^ University of Antwerp, Antwerp, Belgium

## Abstract

**Background:**

We recently developed a CD8 T cell‐induced mouse model that recapitulates definitive hallmarks of Alzheimer's disease (AD) and allowed prediction of previously unknown properties of human sporadic AD (DOI: 10.1073/pnas.2401420121). Identification of a peptide antigen to which the inducing T cells respond in this model (a non‐Aβ epitope on Amyloid Precursor Protein [APP]) allowed us to quantify analogous T cells in human brain and blood, using an APP_471‐479_/Human Leukocyte Antigen(HLA)‐A2 multimer (pHLA) and flow cytometry on blood. Blood levels of these T cells distinguished AD and related MCI from normal aging controls with high accuracy (AUC: 0.883‐0.892), but the assay is currently suitable only for HLA‐A2‐positive (about half of all) patients due to HLA subtype‐restricted binding.

**Method:**

CD8 T cell binding to a non‐self (_ALIAPVHAV_/HLA‐A0201) multimer correlated with APP multimer binding in HLA‐A2‐positive patients (0.42‐0.58x relative to APP/HLA‐A2; r = 0.81; *P* < 0.001). Surprisingly, the same non‐self multimer bound at similar levels to HLA‐A2‐negative patients’ Tcells. We thus quantified levels of age‐related, KLRG1^+^ CD8 T cells binding to this multimer in HLA‐A2‐positive (*n* = 79) and ‐negative (*n* = 44) subjects by flow cytometry from Alzheimer's disease (AD) and normal aging control cohorts, and assessed their correspondence to disease status.

**Result:**

As with the APP pHLA reagent, levels of T cells binding to the non‐self pHLA multimer were significantly diminished in AD irrespective of HLA subtype (*P* < 0.015 by 2‐sided T‐Test). In receiver operating characteristic (ROC) analysis of the HLA‐negative cohort, area under the curve (AUC) was 0.878 for AD (*P* < 0.00001) with 79.4% sensitivity and 98.6% specificity, nearly identical to the APP‐specific CD8 T cells in HLA‐A2‐positive individuals (AUC 0.88; 72.2% sensitivity, 97.2 specificity). Moreover, disease correspondence of the non‐self pHLA‐reactive T cells was improved in HLA‐A2‐positive patients as well (AUC 0.912).

**Conclusion:**

Subtype‐independent pHLA binding by CD8 T cells strongly corresponds to AD status in HLA‐A2‐negative and HLA‐positive individuals, providing a working basis for expanding our AD biomarker assay to all patients. Ongoing work will focus on increasing the sensitivity of HLA‐independent multimer binding to further increase assay utility.

